# Evaluation of the Photocatalytic Activity and Anticorrosion Performance of Electrospun Fibers Doped with Metallic Oxides

**DOI:** 10.3390/polym13122011

**Published:** 2021-06-20

**Authors:** Ainhoa Albistur, Pedro J. Rivero, Joseba Esparza, Rafael Rodríguez

**Affiliations:** 1 Engineering Department, Campus Arrosadía s/n, Public University of Navarre, 31006 Pamplona, Spain; ainhoa.albistur@unavarra.es (A.A.); rafael.rodriguez@unavarra.es (R.R.); 2 Institute for Advanced Materials and Mathematics (INAMAT2), Campus Arrosadía s/n, Public University of Navarre, 31006 Pamplona, Spain; 3 AIN, Asociación de la Industria Navarra, 31191 Pamplona, Cordovilla, Spain; jegorraiz@ain.es

**Keywords:** electrospun fiber mat, polyacrylic acid (PAA), β-cyclodextrin (β-CD), thermal crosslink, corrosion protection, photocatalytic activity testing

## Abstract

This paper reports the development and characterization of a multifunctional coating that combines anticorrosion and photocatalytic properties, deposited by means of the electrospinning technique. In the first step, a functional electrospun fiber mat composed of poly(acrylic acid) (PAA) and β-cyclodextrin (β-CD) was obtained, showing high water insolubility and great adhesion increased by means of a thermal crosslinking process (denoted as PAA + β-CD). In the second step, the fibers were doped with particles of titanium dioxide (denoted as PAA + β-CD/TiO_2_) and titanium dioxide plus iron oxide (denoted as PAA + β-CD/TiO_2_/Fe_2_O_3_). The morphology and fiber diameter of the electrospun mats were evaluated by using confocal microscopy, whereas the presence of the metal oxides in the electrospun fibers was corroborated by scanning electron microscopy (SEM) and X-ray fluorescence (XRF), respectively. In addition, electrochemical tests in saline solution revealed that the sample composed of PAA + β-CD/TiO_2_/Fe_2_O_3_ showed the highest corrosion protection efficiency of all the samples, which was directly associated to lower corrosion current density and higher corrosion potential. Furthermore, the paper reports a novel approach to in situ determination of methylene blue (MB) degradation onto the coating. The results revealed complete degradation of MB, which is perfectly appreciated by total discoloration of the film in the irradiated zone (from bluish to a white spot). The main conclusions of this research are the efficiency of the electrospun system PAA + β-CD/TiO_2_/Fe_2_O_3_ for developing photocatalytic activity and corrosion protection and the utility of the dry MB discoloration tests to evaluate photocatalytic activity.

## 1. Introduction

Nowadays, the implementation of surface engineering techniques with a high specific surface area is a challenging topic in the nanotechnology field thanks to the possibility of designing functionalized surfaces with advanced properties in a wide variety of industrial applications [1,2]. In this sense, the electrospinning technique is a very interesting approach due to its scalability, versatility and potential uses for the development of new functional nanofiber materials [3,4] which can be obtained from numerous polymeric materials with different nature (water solubility, water insolubility or biocompatibility) [5], showing potential applications in tissue engineering [6], electronic devices [7], wound healing [8], drug delivery [9], organic pollutants degradation [10], enzyme immobilization [11], air filtration [12], catalysis [13] or antibacterial surfaces [14], among others. In addition, the electrospinning technique has attracted attention due to the possibility of obtaining continuous fibers with the desired morphology, exhibiting outstanding performance, like high porosity combined with a large surface-to-volume ratio [15,16]. A standard electrospinning device consists of three major components: a high-voltage power supply, a syringe with a control pump connected to a needle to which high voltage is supplied and a grounded collector. An electrically charged polymer solution is stretched and refined in a high-voltage electrostatic field by solvent volatilization and becomes a micro-nano-level fiber with a certain accumulation density [17,18]. In this process, once high voltage is applied, the polymeric solution located at the tip of syringe is electrostatically charged, forming a cone-like structure known as “Taylor cone” [19]. Good control in the electrospun fiber morphology can be modulated as a function of the operational parameters (flow rate, applied voltage or tip to collector distance) [20] as well as the resultant intrinsic polymeric precursor properties (concentration, viscosity, surface tension, conductivity, nature of solvent) [21]. Another important aspect is that the electrospinning technique can be implemented for precursors of different nature, such as water soluble, water insoluble or even biocompatible polymers.

This process is being studied by the scientific community as a promising scalable method for the design of environmental applications which can be potential alternatives to conventional methods [22]. In this sense, among all the degradation processes, photocatalytic technology is considered as a powerful and advanced oxidative process (AOP), enabling the generation of strong oxidant species and radicals which can induce many reactions for the degradation of organic compounds [23,24]. More specifically, the photocatalytic process takes place upon the activation of a semiconductor with electromagnetic radiation from sunlight or artificial light. This semiconductor can absorb photons with sufficient energy to inject an electron from the valence band (VB) to the conduction band (CB) and, as a result, the generation of electron-hole (e^−^h^+^) pairs can be obtained [25,26]. Another aspect to remark is that among all the materials used for catalysis, titanium oxide (TiO_2_) has gained great popularity due to its excellent properties (i.e., long-term stability, high capacity for oxidation resistance, low toxicity or low preparation process) under ultraviolet (UV) light radiation and is being extensively used as a reference material in the field of photocatalysis [27,28,29] and self-cleaning [30]. In addition, it has been demonstrated that its corresponding photoactivity is intrinsically associated to several key factors such as crystallinity, morphology and surface area [31,32]. Due to these special features, the use of porous materials with a high surface area and a high aspect ratio obtained by the electrospinning technique as supporting materials combined with good immobilization of TiO_2_ nanoparticles into the electrospun fibers has gained enormous interest in the field of photocatalysis [33]. However, because the classical photocatalyst of TiO_2_ nanostructures is a wide-bandgap semiconductor (3.0–3.2 eV), TiO_2_ can only absorb in the ultraviolet (UV) region (λ < 380 nm), which accounts for 3–5% of the solar spectrum [34]. Due to this, the development of narrow-bandgap semiconductors as photocatalysts is an emerging hot topic in the area of solar-to-fuels conversion for hydrogen generation. Very interesting work is presented in [35], where lacunary Keggin-type phosphotungstates (PW9) clusters have been successfully loaded onto g-C3N4 nanosheets (NSs), making the formation of heterojunction NSs possible via a facile self-assembly process due to the electrostatic interaction which enables an enhancement in the photocatalytic H_2_ generation. Other alternatives are based on the use of visible light-responsive photocatalysts which can be also employed for photocatalytic hydrogen evolution [36,37].

By using the electrospinning technique, representative work is presented in [38], where a novel flexible photocatalyst of AgBr/BiOBr/polyacrylonitrile (PAN) composite mats (CMs) is developed through controllable assembly of AgBr/BiOBr nano-heterostructures on electrospun polyacrylonitrile nanofibers (PAN NFs) via a three-step synthesis route. In this work, upon visible light irradiation, enhancement in photocatalytic activity has been observed, showing excellent separability during the degradation of methyl orange in water. Other work is focused on the fabrication of composite nanofibers into a polymeric matrix by varying the composition of electrospinning solutions based on the combination of TiO_2_ with other precursors such as silver nanoparticles (AgNPs) [39], gold nanonorods (AuNRs) [40] or other different metallic oxides [41], making enhancement in the resultant photocatalytic properties possible. In addition, thanks to the great versatility of the electrospinning technique and the possibility of incorporating a wide variety of different precursors, it is possible to design multifunctional surfaces which can be perfectly extrapolated to other research lines such as the design of highly efficient antibacterial surfaces [42].

The combination of different oxides has been explored by several authors as a strategy to extend the range of photocatalytic activation to larger wavelengths. In particular, different iron oxides associated to titanium dioxide have shown enhanced photocatalytic performance [43,44] as well as good stability [45,46]. In this work, we presented a new one-step electrospinning process based on the synergetic effect of combining iron oxide (Fe_2_O_3_) and titanium dioxide (TiO_2_) into an insoluble polymeric electrospun supporting matrix. The experimental results have demonstrated simultaneous enhancement in photocatalytic performance under both UV and visible light, which was corroborated by the degradation of a dye pollutant (methylene blue, MB), as well as in the resultant corrosion resistance. Finally, these findings, associated to this specific surface area morphology, can be used as an efficient eco-friendly and economical alternative for its implementation on a real industrial scale.

## 2. Experimental Section

### 2.1. Reagents and Materials

Poly(acrylic acid) (PAA; *M_w_* ≈ 450,000), β-cyclodextrin (β-CD, purity 98%), ethanol (96%), titanium (IV) oxide (TiO_2_, anatase nanopowder), iron oxide (Fe_2_O_3_) particles and methylene blue (MB) were provided from Sigma-Aldrich (St. Louis, MO, USA). All the reagents were used without any further purification. Firstly, the electrospun fibers were deposited onto standard glass slides for complete characterization of adhesion, morphological and photocatalytic properties. Secondly, the electrospun fibers were also deposited on metallic substrates (austenitic stainless steel, AISI 304) for the characterization of the resultant corrosion properties by using a sodium chloride aqueous solution (3.5 wt% NaCl) from Sigma-Aldrich (St. Louis, MO, USA) as electrolytic corrosive medium.

### 2.2. Electrospinning Procedure

**Figure 1:**
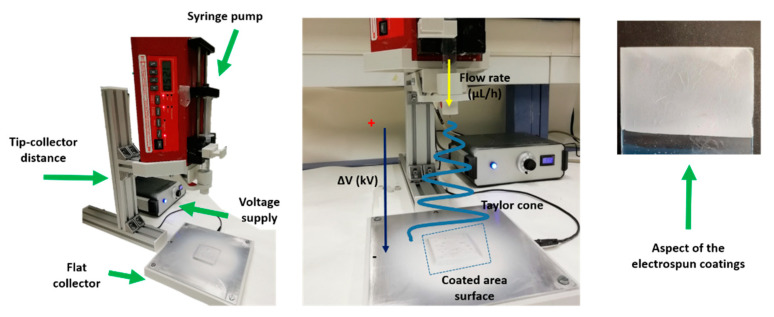
Experimental setup used for the fabrication of the electrospun fibers as a function of the control of tip–collector distance, applied voltage and flow rate, showing the aspect of the electrospun fibers.

Strict control of three specific parameters, flow rate (µL/h), applied voltage (kV) and tip-collector distance (cm), is performed for adequate formation of the Taylor cone, enabling the formation of successfully electrospun fibers onto the reference substrate [4,21,47]. The base spinning solution was prepared by dissolving 0.8 g of PAA and 0.128 g of β-cyclodextrin in 11.6 mL of ethanol, which was magnetically stirred for 24 h under room temperature until a well-dispersed solution was achieved. After that, 0.5 g of TiO_2_ and a mixture of 0.45 g of TiO_2_ and 0.02 g of Fe_2_O_3_ were added and stirred for 24 h to get PAA + β-CD/TiO_2_ and PAA + β-CD/TiO_2_/Fe_2_O_3_ homogeneous solutions. A 20-gauge needle with an inner diameter of 0.6 mm was used for the fabrication of the electrospun coatings. A voltage of 15 kV was applied for the electrospinning process, and the tip–collector distance was fixed at 20 cm with a flow rate of 1.3 mL/h. Finally, a schematic representation of the electrospinning setup used for the fabrication of the electrospun fibers is presented in Figure 1, showing the corresponding aspect of the obtained electrospun coatings.

### 2.3. Coating Adhesion Measurements

Adhesion performance between electrospun coating and reference metallic substrate (austenitic stainless steel, AISI 304) was analyzed according to the ASTM D3359, where an X-cut and the corresponding criteria of adhesion rating are evaluated [48,49]. Specifically, using a scalpel, two cuts that fully penetrated the coating down to the substrate were made in order to obtain an X with 30–45° angle between the cuts. Then, pressure-sensitive tape was applied onto the center of the intersection of the cuts, and firm pressure was applied to ensure adequate adhesion of the tape to the electrospun coating. Finally, after an application time of 120 s, the tape was removed by pulling it off rapidly, and the resultant X-cut area was inspected in accordance with scale classification. The adhesion rating ranges from 0 up to 5, with an increasing value of 1, assigning 0 and 5 to the lowest and highest adhesion values, respectively.

### 2.4. Morphology, Wettability and Chemical Characterization

The surface morphology of the electrospun coatings was analyzed by using confocal microscopy (model S-mart, SENSOFAR METROLOGY, Barcelona, Spain) and applying a course shift single algorithm with an objective of EPI 50X v35 for a final area of 340.03 × 283.73 μm. These measurements were obtained with three different filters: low filter (F-operator-level), high filter (S-filter, standard cut off λ_s_: 2.5 µm) and Gaussian filter (L-filter, standard cut off λ_c_: 0.08 mm, Sa < 0.02 µm), with the aim of evaluating the resultant average fiber diameter after a total of 30 measurements for each sample of study. In addition, the surface morphology was also analyzed by using Field Emission-Scanning Electron Microscopy (FE-SEM Hitachi S4800, Tokyo, Japan). Additionally, by using an electron dispersive X-ray analysis (EDX) microprobe and backscattered electron images, the distribution of the different elements was determined. Finally, the chemical composition of the samples was analyzed by X-ray fluorescence (XRF) using FISCHERSCOPE X-RAY XDAL FD (HELMUT FISCHER GMBH, Stuttgart, Germany) equipment. 

X-ray diffraction (XRD) analysis was performed to corroborate the crystallographic peaks of the nanoparticles. A Bruker D8 Discover machine equipped with a Cr Kα radiation line (λKα1 = 2.29 Å; 40 kV; 40 mA) under Bragg–Brentano configuration was used. A scan covering 2ϴ angles from 20° to 130° at a scan rate of 0.02° every 5 s was performed on each sample.

A contact angle meter (CAM 100 KSV Instruments, Burlington, VT, USA) was used for the evaluation of the wettability of the electrospun coatings. The static contact angle was obtained by analyzing the captured images using the tangent method algorithm. The average value of the contact angle between the sample surfaces and a minimum of five deionized water drops was measured using the sessile drop method.

### 2.5. Electrochemical Measurements

The electrochemical measurements related to the Tafel polarization curves were carried out by using an Autolab Potentiostat/Galvanostat PGSTAT302N (Metrohm, Herisau, Switzerland) and were performed at room temperature in 3.5 wt% NaCl aqueous solution by using a conventional three-electrode cell consisting of a working electrode (stainless steel), a silver chloride Ag-AgCl reference electrode and a platinum counter electrode. As an initial step, before conducting the electrochemical measurements, all the electrospun samples were immersed in the corrosion media for a period of time of 30 min to make sure that the system was in steady state with the aim of stabilizing the open circuit potential (OCP). The Tafel polarization measurements were obtained by scanning the electrode potential automatically from −500 to +500 mV with respect to the OCP voltage at a scan rate of 2 mV·s^−1^. The corrosion properties were calculated by using Tafel slope analysis by establishing a relationship between current density and electrode potential during the polarization test. The corrosion data were obtained from Tafel polarization curves, where it was obtained by superimposing a straight line on the linear portions of the cathodic and anodic curves. Other corrosion parameters, such as equivalent weight of the metal, density or exposed surface area, were also required as input parameters. Finally, as a result, the software generated the complete set of corrosion parameters, and the resultant corrosion rate was calculated according to Equation (1) [50,51]:

(1) $$Corrosion\;rate=327\times \frac{I_{corr}\cdot M}{V\cdot D\cdot A}\times 100\%$$

where 327 = 1 year (in seconds)/96,500, and 96,500 = 1 F in coulombs. *I_corr_* is the corrosion current, which is determined by the intersection of the linear portions of the anodic and cathodic sections of the Tafel curves, *M* is the atomic mass, *V* is the valence (number of electrons that are lost during the oxidation reaction), *D* is the density and *A* is the exposed area of the sample.

Finally, the corrosion protection efficiency (*η*) from Tafel polarization curves was calculated from Equation (2) by using the same methodology as [52,53], where *I_corr_* and *I_corr_* (*C*) correspond to the corrosion current densities of the bare AISI 304 substrate and the electrospun coated samples, respectively.

(2) $$\eta \;\left(\%\right)=\frac{I_{corr}-I_{corr}\;\left(C\right)}{I_{corr}}\times 100\%$$

### 2.6. Photocatalytic Activity of Electrospun Fibers

**Figure 2:**
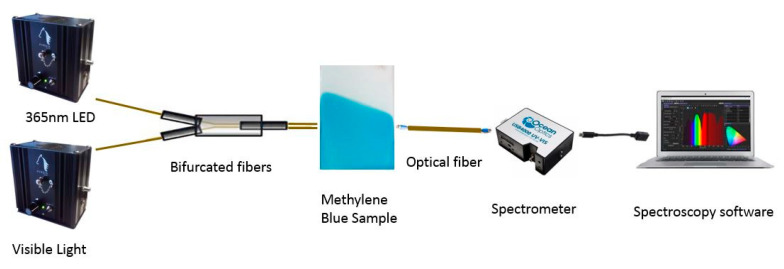
Experimental setup used to monitor the photocatalytic efficiency of the electrospun fibers doped with a methylene blue solution after irradiation with both 365 nm LED and visible light.

First of all, once the electrospun coatings were fabricated as a function of the input parameters of flow rate, applied voltage, tip–collector distance and deposition time, the samples were immersed in a methylene blue solution (10 mg/L) for 24 h with the aim of obtaining a clearly bluish coating that is clearly indicative that methylene blue (MB) has been successfully incorporated into the electrospun PAA fibers. In order to monitor the photocatalytic activity of the electrospun samples against MB, an optical transmission setup was used (Figure 2). This setup consisted of two light sources, a UV radiation Light Emission Diode (LED, λ = 365 nm) and a white halogen visible light source, which were connected to a bifurcated fiber, and the resultant coupled light passed through the bluish electrospun samples that were connected to one end of an optical fiber in the UV–Vis spectrometer (Ocean Optics HR4000, Ocean Insight, FL, USA). Finally, this spectrometer was connected to a PC in order to obtain the spectral data in the range between 550 and 750 nm. The resultant photocatalytic performance analysis was carried out by degrading MB films under continuous irradiation for a final period of 12 h for all the samples of study.

## 3. Results and Discussion

In this Section, firstly, the influence of thermal treatment on the improvement of coating adhesion of the PAA fibers to the substrate is analyzed. Secondly, the effect of incorporating inorganic metal oxide nanoparticles into the PAA fibers is evaluated with the aim of showing enhancement in photocatalytic activity and corrosion resistance.

### 3.1. Coating Adhesion

**Figure 3:**
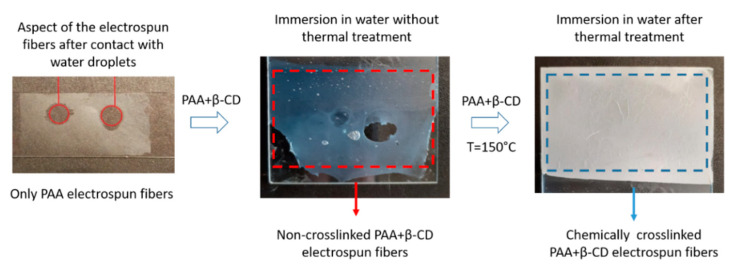
Aspect of the electrospun fiber coatings after immersion in water for PAA only, PAA + β-CD without thermal treatment and thermally treated PAA + β-CD electrospun fiber mats with the corresponding change in the coloration induced by the chemical crosslink.

First of all, an important aspect to remark is that the deposition of PAA fibers only onto the reference substrates without the presence of β-CD produced easily removable electrospun fibers after immersion in ultrapure water. Due to this, the addition of a crosslinker agent such as β-CD enables the fabrication of highly crosslinked PAA fibers with a high degree of insolubility after long-term water immersion [54]. In order to obtain this water stability, it is necessary to induce a thermal crosslink at an elevated temperature of 150 °C for 30 min, making the formation of ester bonds by an esterification process possible [55]. In Figure 3, a schematic representation of the resultant evolution of the aspect of the electrospun fibers (only PAA, PAA + β-CD and thermally treated PAA + β-CD fibers) after immersion in ultrapure water is shown. It can be clearly appreciated that thermal treatment of the PAA + β-CD fibers induced the formation of highly crosslinked fibers with a change in the resultant aspect from slightly transparent to white-colored, showing better water stability, because the thermally treated electrospun fibers were not removed from the substrate after long-term water immersion. A similar approach, based on the application of a specific thermal treatment for obtaining highly crosslinked fibrous mats, is presented in [56] by using poly(vinyl alcohol) (PVA) and poly(acrylic acid) (PAA) as electrospun polymeric precursors.

**Figure 4:**
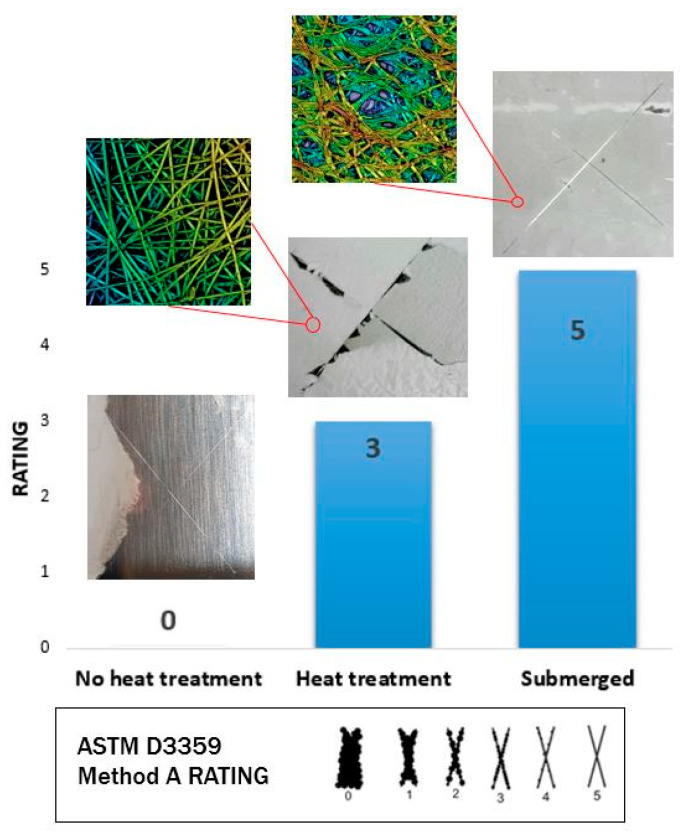
Aspect of the X-cut adhesion tests for the electrospun PAA + β-CD before thermal treatment (rating 0), after thermal treatment (rating 3) and after combining both thermal treatment and water immersion (rating 5) according to ASTM D3359 Method A Rating.

Once this enhancement in the resultant water stability was confirmed, it was also demonstrated that this heat treatment also induced an increase in the adhesion of the fiber mats onto the underlying substrate [57,58,59], which is a key point for further implementation in industrial applications. In this work, the resultant coating adhesion performance was analyzed according to ASTM D3359 as a function of the area removed by the tape, as it can be appreciated in Figure 4. An important aspect to remark is that no changes in coating adhesion were observed up to temperatures close to the glass transition of the polymeric precursor of PAA. As an initial experiment, when the fibers were thermally treated at 100 °C, a clear adhesion improvement was observed at a glance. Before the heat treatment, the fibers were easily removed when the X-cut adhesion test was performed. However, after thermal treatment at 100 °C, the X-cut adhesion tests showed that the adhesion between the heat-treated electrospun coating and the aluminum substrate was still poor, as more than 50% of the area was removed, showing a rating value of 1 according to the ASTM D3359 Method A Rating. Due to this, by increasing the temperature up to 150 °C, an important improvement into a rating value of 3 was observed, which was directly associated to the chemical crosslink previously commented.

From these results, it can be clearly observed that non-thermally treated PAA + β-CD fibers showed the lowest adhesion properties (rating value of 0), because complete removal of the electrospun polymeric film was obtained. However, a complete change in the adhesion value was observed after thermal treatment at 150 °C, because the sample showed a lower amount of removed area (rating value of 3). Finally, a surprising result was observed when the sample had been immersed in ultrapure water (24 h immersion), because a considerable improvement of the adhesion coating was clearly presented, showing the best adhesion properties without any amount of removal of the electrospun coating (rating value of 5). This high value is associated to the weak polyelectrolyte nature of the polymeric precursor of PAA, which can display a high density of carboxylic acid groups and negative charges, making possible a complex balance of interactions (mostly electrostatic and van der Waals interactions) that can also promote strong adhesion onto the substrate [60]. Finally, a total change in the appearance of the electrospun fibers was also appreciated and associated to the pH-sensitive swelling behavior of PAA after immersion in ultrapure water [61,62,63].

### 3.2. Morphology, Wettability and Chemical Characterization

**Figure 5:**
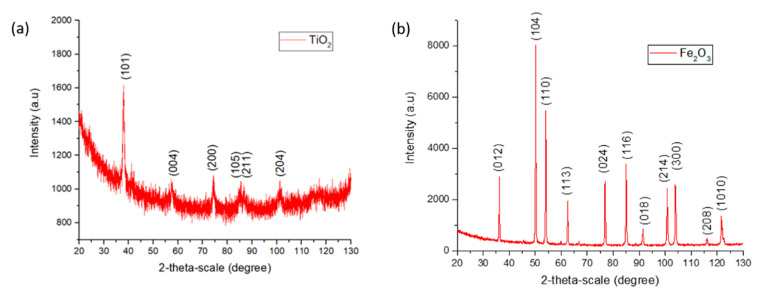
XRD of TiO_2_ (**a**) and Fe_2_O_3_ (**b**) nanoparticles.

**Figure 6:**
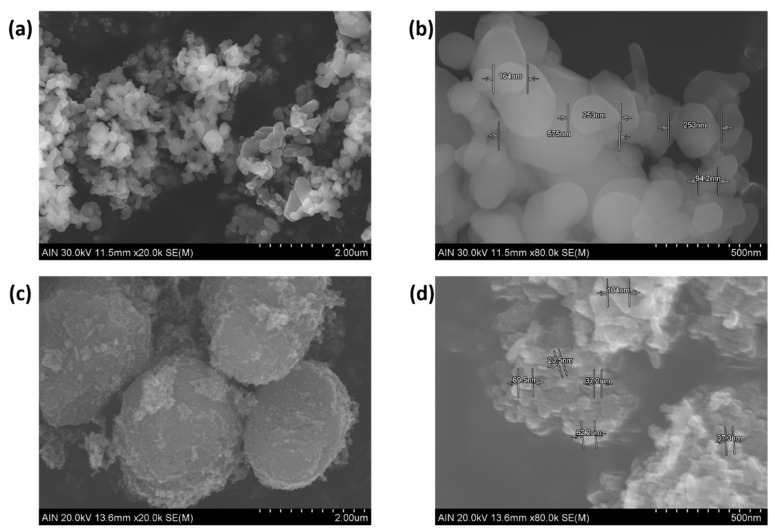
SEM images for Fe_2_O_3_ (**a**,**b**) and TiO_2_ (**c**,**d**) nanoparticles.

The nature and crystallinity of TiO_2_ and Fe_2_O_3_ NPs in the electrospun coatings was analyzed by XRD (see Figure 5). In addition, SEM images of the pure nanoparticles before they were incorporated into the fibers are presented in Figure 6. Compared to Fe_2_O_3_, TiO_2_ nanoparticles showed smaller particle size and higher aggregation degree.

**Figure 7:**
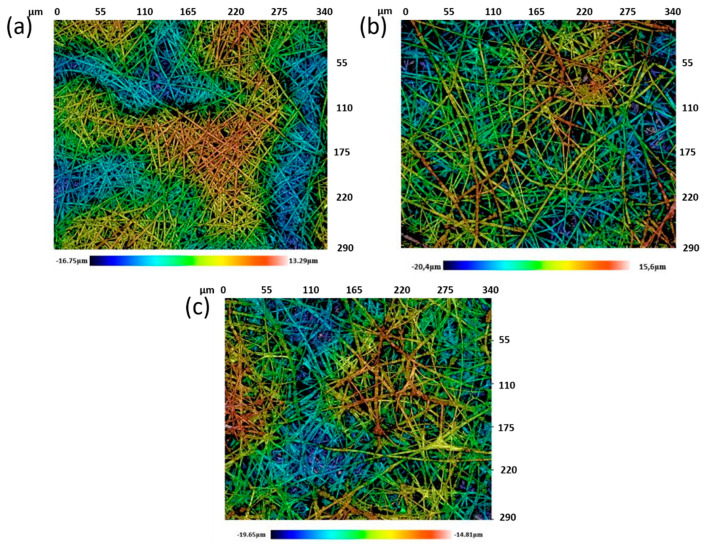
Confocal images of the samples of study for crosslinked electrospun PAA + β-CD fibers (**a**), PAA + β-CD + TiO_2_ fibers (**b**) and PAA + β-CD + TiO_2_ + Fe_2_O_3_ fibers (**c**).

The resultant surface morphology of the electrospun coatings was deeply studied in order to show any differences in the resultant fiber diameter by using a confocal microscope. Figure 7 shows the confocal images of the different samples of study.

From these confocal images, it can be seen that a fibrous mat with a porous fibrous structure was obtained with metal oxide precursors uniformly distributed onto the surface of the crosslinked electrospun fibers. In addition, as an interesting result, the mean diameter of the only polymeric fibers (PAA + β-CD) was the lowest (2.05 ± 0.36 µm), whereas the addition of the metal oxide precursors produced an increase in the resulting diameter for PAA + β-CD + TiO_2_ (2.2 ± 0.73 µm) and PAA + β-CD + TiO_2_ + Fe_2_O_3_ (3.16 ± 0.67 µm).

**Figure 8:**
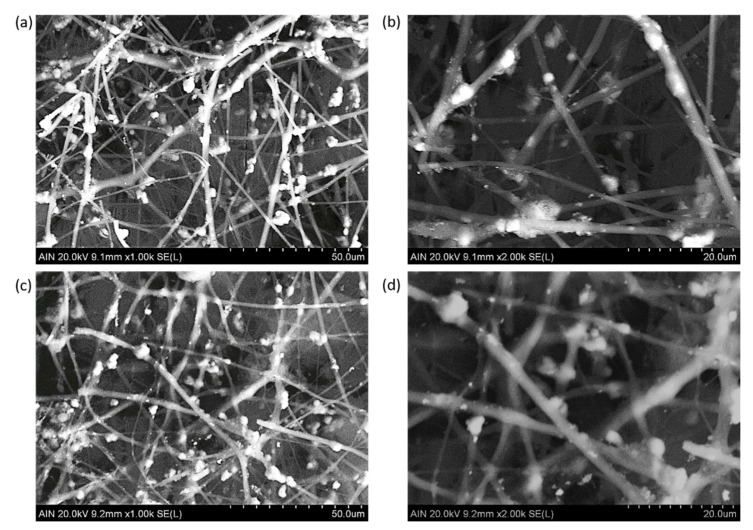
SEM images of the samples composed of PAA + β-CD + TiO_2_ fibers (**a**,**b**) and PAA + β-CD + TiO_2_ + Fe_2_O_3_ (**c**,**d**) fibers at different scale bars of 50 µm (**a**,**c**) and 20 µm (**b**,**d**).

According to this, it can be concluded that a higher amount of these precursors was grafted in the fiber surface and contributed to a larger fiber diameter [56]. In addition, SEM images were also made in order to corroborate the presence of the resultant metallic oxide precursors in the electrospun fibers (see Figure 8). The particles with a bright color are perfectly distributed onto the polymeric fiber mats. In addition, the variation of the water contact angle (WCA) of the different electrospun samples was also analyzed. As an interesting result, all the electrospun samples have shown intrinsic superhydrophilic behavior with contact angle values less than 5° for contact time of 60 s. When a polymeric precursor with polar functional groups (hydroxyl groups), such as PAA [64], is used, the resulting surface roughness associated to the electrospinning process and the external distribution of the metallic oxide nanoparticles onto the porous fiber mat (corroborated by the SEM images) with clear superhydrophilic behavior [65] makes this wetting state possible.

**Figure 9:**
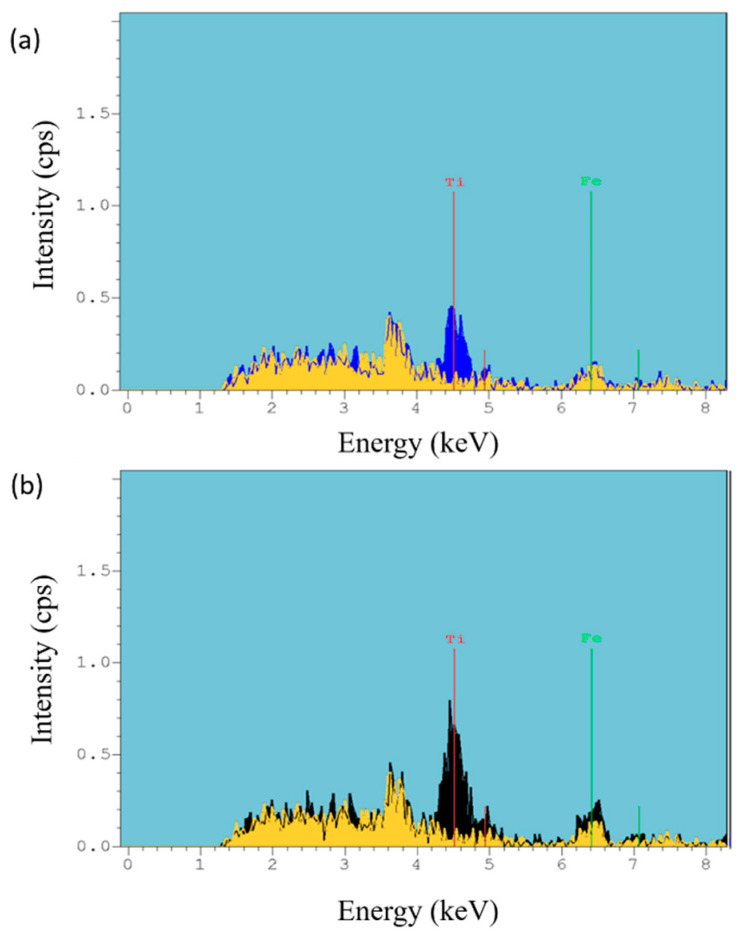
Comparative X-ray fluorescence of the electrospun coatings for PAA + β-CD versus PAA + β-CD + TiO_2_ (**a**) and for PAA + β-CD versus PAA + β-CD + TiO_2_ + Fe_2_O_3_ (**b**).

Finally, in order to corroborate the chemical composition of the outer surface, X-ray fluorescence was also performed. Figure 9 shows a comparative X-ray fluorescence image between only polymeric electrospun sample (PAA + β-CD) and samples composed of PAA + β-CD + TiO_2_ (Figure 9a) and PAA + β-CD + TiO_2_ + Fe_2_O_3_ (Figure 9b), where the location of the peaks related to Ti (4.5 keV) and Fe (6.4 keV) can be clearly observed with high intensity in comparison to that of the only polymeric electrospun sample.

### 3.3. Corrosion Evaluation

An aspect to remark is that electrospun nano/microfibers deposited onto metallic substrates (aluminum, magnesium, steel) with intrinsic superhydrophobic or even highly hydrophobic behavior give long-term protection from corrosion [66]. In this sense, representative works can be found by using polyvinyl chloride (PVC) [67], polystyrene (PS) [68], poly(vinylidene fluoride) (PVDF) [69] or even perfluorinated block copolymer [70], among others. However, the use of metallic oxide nanoparticles combined with these synthetic hydrophobic polymeric precursors can be also employed as a novel approach to the development of highly anticorrosive surfaces. As a representative example, in comparison with only polymeric samples, the use of zinc oxide nanoparticles (ZnO NPs) embedded into these electrospun fibers has offered long-term corrosion resistance [59,71,72], although other types of metal oxide nanoparticles such as alumina (Al_2_O_3_) [73], silica (SiO_2_) [74] or titanium dioxide (TiO_2_) [75] endowing the design of highly porous superhydrophobic coatings with superior corrosion resistance in comparison with that of a pure polymeric precursor.

**Figure 10:**
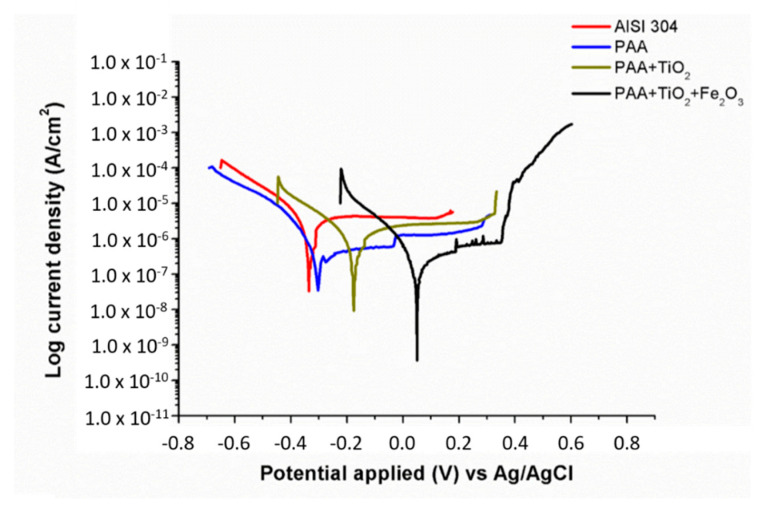
Tafel plots for the reference AISI 304 substrate and for the different samples coated with electrospun fibers in 3.5 wt% NaCl aqueous solution.

In this work, the effect on corrosion resistance of the respective metallic oxide precursors embedded into the electrospun fibers was evaluated by using potentiodynamic polarization curves in a chloride corrosive solution (3.5 wt% NaCl). The Tafel curves of all the samples of study are presented in Figure 10, whereas the related parameters (i.e., corrosion potential, current density or corrosion rate, among others) are shown in Table 1. First of all, it is well-known that an improvement in corrosion resistance can be estimated by controlling the corrosion potential and the current density. More specifically, an increase in corrosion potential or a decrease in current density are directly associated with an enhancement in corrosion properties [76,77], although corrosion potential is considered as one of the most crucial factors in the resultant corrosion rate as well as in surface susceptibility [78,79]. This aspect is in concordance with the experimental results where corrosion potential (E_corr_) values gradually decreased from the bare reference substrate up to the electrospun samples, ranging from −0.32544 (AISI 304) to 0.046167 V (PAA/TiO_2_/Fe_2_O_3_). First of all, no differences were found between the reference substrate (stainless steel AISI 304) and only polymeric coating (AISI 304 + PAA), with both samples showing the lowest corrosion potential with similar values of current density and corrosion rate. This result is in concordance with the superhydrophilic behavior of the PAA sample, due to which no corrosion protection was observed in this sample. However, an aspect to remark is that the addition of TiO_2_ NPs into the PAA electrospun fibers produced a reduction in the corrosion potential (E_corr_) value from −0.30467 (only PAA) up to −0.16752 V (PAA/TiO_2_). The sample composed of PAA/TiO_2_/Fe_2_O_3_ exhibited more than ten times lower corrosion current density in comparison with that of the bare substrate. From these experimental results, it can be corroborated that the combination of these two metallic oxide precursors (TiO_2_ + Fe_2_O_3_) into the PAA electrospun fibers was more effective in improving the resultant corrosion resistance by showing the highest corrosion potential (0.046167 V) and the lowest current density of all the samples of study. In addition, the resultant corrosion rate of this sample was the lowest of all the samples of study, showing the highest corrosion protection efficiency (90.9052%) in comparison with that of PAA (no protection) and PAA/TiO_2_ (28.6294%). Finally, this excellent corrosion resistance was obtained thanks to a lower corrosion rate, which was directly associated to higher corrosion potential and lower current density.

### 3.4. Photocatalytic Efficiency

**Figure 11:**
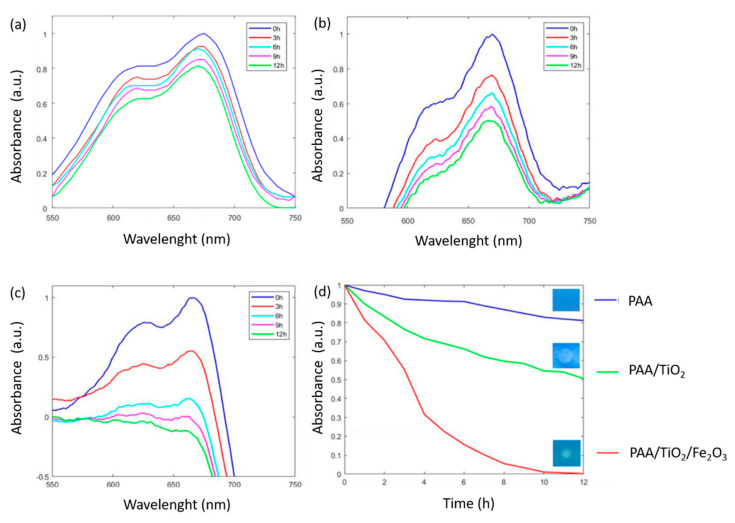
UV-Vis spectra changes of the MB-doped electrospun samples after UV-Vis irradiation for PAA + β-CD (**a**), PAA + β-CD + TiO_2_ (**b**), PAA + β-CD + TiO_2_ + Fe_2_O_3_ (**c**) and a comparative evolution of all the samples of study with the final aspect of coatings after 12 h of irradiation (**d**).

In order to evaluate the photocatalytic activity of the coatings, all the electrospun samples were immersed in a methylene blue (MB) solution, showing a characteristic bluish coating with a maximum wavelength (λ_max_) at 665 nm. After that, the photocatalytic activity of the metal oxides immobilized in the electrospun fibers was evaluated as a function of MB decoloration under both UV and visible radiation light (setup in Figure 2) at ambient room temperature. Figure 11 shows the spectral variation changes in MB concentration with the interaction of each type of nanofibers as a function of irradiation time. The initial conclusion derived from this test was that only the PAA + β-CD electrospun mat (Figure 11a) did show any decoloration of MB, indicating that the oxideless electrospun polymeric fiber mats had no contribution to the decoloration of the dye. However, a significant change in photocatalytic activity of PAA + β-CD + TiO_2_ (Figure 11b) was appreciated, where a decolorization of MB was clearly observed during the irradiation time. The mechanism of TiO_2_ photocatalysis is associated with the photoinduced generation of electron-hole pairs on the TiO_2_ surface [80], making possible the generation of significant reactive oxygen species (ROS) that directly oxidize the MB dye with the corresponding decoloration [33,43]. In addition, another interesting aspect is that β-CD can act as an efficient host-guest molecule for the entrapment of dye molecules in its cavity [44], making possible an enhancement of the interaction between TiO_2_ and the colorant. However, this effect was most relevant in the PAA + β-CD + TiO_2_ + Fe_2_O_3_ sample (Figure 11c), where a more rapid decoloration of the MB dye was observed, showing a clear total decoloration after 12 h (see inset in Figure 11d). In this sample, a synergetic effect of both TiO_2_ and Fe_2_O_3_ was created, because dye molecules entered into the cavity of β-CD, which was linked to the metal oxide surface in the equilibrium stage, absorbing more light radiation, followed by a further excitation [41]. This measurement of photocatalytic activity directly on tinted dry coating is one of the principal novelties of this paper, because most previous investigations carried out this evaluation by dispersing particles or fibers in an MB solution [81].

After observing these results, it can be clearly appreciated that the rate of degradation of MB under both UV and visible radiation was considerably enhanced when the combination of both metal oxides inside the polymeric PAA + β-CD electrospun fiber mat was used, in comparison with that of the electrospun sample composed of TiO_2_ only.

**Figure 12:**
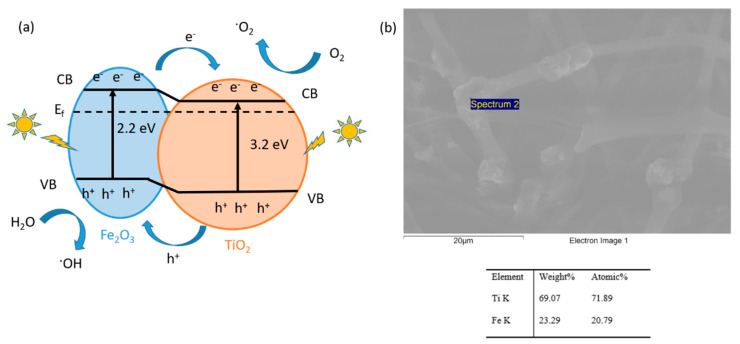
(**a**) Schematic diagram of the photocatalytic mechanism; (**b**) SEM image with EDX mapping in the selected area indicating the presence of both types of nanoparticles for the heterojunction mechanism.

This result can be derived from the small band gap of the Fe_2_O_3_ metal oxide precursor, which can increase the visible light absorption of TiO_2_, and the Fe_2_O_3_/TiO_2_ heterojunction that can effectively improve the lifetime of the photogenerated electron-hole pairs [82]. A schematic diagram showing the corresponding band configuration and the electron-hole separation at the interface of the TiO_2_/Fe_2_O_3_ heterojunction can be clearly observed in Figure 12. Under UV-Vis light irradiation, both TiO_2_ and Fe_2_O_3_ can be photoexcited to produce electron/hole (e−/h+) pairs [83]. As a promising result, the corresponding interfacial e−/h+ separation could be directional thanks to the possibility of generating reactive oxygen species (O_2_) and hydroxyl radicals (OH) with high activities that degrade MB rapidly. In order to obtain these reactive species, on the one hand, the photogenerated electrons on the conduction band (CB) of Fe_2_O_3_ are transferred to the CB of TiO_2_ with a lower potential, and these electrons are captured by the adsorbed oxygen, making possible the production of reactive oxygen species. On the other hand, the holes remaining in the valence band (VB) of TiO_2_ are transferred to the VB of Fe_2_O_3_ with higher potential, and these holes could be trapped by H_2_O molecules to form strong oxidizing hydroxyl radicals [84]. In addition, in order to corroborate this heterojunction mechanism, the SEM image with EDX analysis indicated the presence of both types of nanoparticles in the selected spectrum area. Finally, this considerable enhancement of photocatalytic activity of the PAA + β-CD + TiO_2_ + Fe_2_O_3_ sample was mainly attributed to this dual positive effect associated to the heterojunction structure combined with the use of cyclodextrin as a host-guest molecule that can facilitate the electron injection from the excited dyes to the metal oxide conduction band, with the corresponding improvement in degradation.

There are still some challenges that we consider relevant for the continuation of this research line. The most important are the optimization of coating efficiency by tuning the best combination of fiber diameter and density, the extension of this research to other wavelengths and dyes in order to explore other ranges of photocatalytic behavior as well as adapting this new testing method to measure the stability of the system, which implies the calibration of successive tinting and decoloring processes.

## 4. Conclusions

In this work, the fabrication of electrospun fiber mats with a high degree of insolubility was obtained as a function of a thermal crosslink curing step, showing great adhesion onto the reference substrate. The immobilization of metal oxide precursors such as titanium dioxide (TiO_2_) and iron oxide (Fe_2_O_3_) was successfully performed, where the polymeric precursors (PAA + β-CD) acted as an efficient host-guest matrix. The photocatalytic activity under UV-Vis light as well as the corrosion resistance of the electrospun fiber mat coatings were evaluated for three different samples of study: PAA + β-CD (only polymeric matrix), PAA + β-CD + TiO_2_ and PAA + β-CD + TiO_2_ + Fe_2_O_3_. The obtained experimental results have demonstrated the synergetic effect of both TiO_2_ and Fe_2_O_3_ onto the electrospun fibers, because the highest corrosion protection efficiency was obtained (ŋ = 90.9052%), which was in concordance with the highest corrosion potential (0.046167 V) and the lowest current density of all the samples of study. By using a new strategy to evaluate photocatalytic activity directly on a tinted dry coating, we have demonstrated that the sample composed of both metal oxides also showed the best photocatalytic performance, which was attributed to the use of β-CD as a host-guest molecule for the entrapment of dye molecules in its cavity, making possible the electron injection from the excited dyes to the metal oxide conduction band, with the corresponding improvement in MB degradation rate.

## Figures and Tables

**Table 1:** The electrochemical parameters from Tafel polarization curves for bare stainless steel (AISI 304) and the coated reference substrate with all the electrospun coatings in 3.5 wt% NaCl aqueous solution.

Sample	β_a_ (mV/dec)	β_c_ (mV/dec)	*i*_corr_ (µA)	*E*_corr_ (V)	Corrosion Rate (mm/year)	Corrosion Protection (%)
Bare AISI 304	0.050031	0.033897	0.43735	−0.32544	0.0050005	-
PAA	1.6319	0.13479	0.43712	−0.30467	0.0050793	0.0052589
PAA/TiO_2_	0.088208	0.055193	0.31214	−0.16752	0.0035718	28.6294
PAA/TiO_2_/Fe_2_O_3_	0.042805	0.028691	0.039776	0.046167	0.00046219	90.9052

## Data Availability

Data sharing not applicable.
